# A Tangible Solution for Hand Motion Tracking in Clinical Applications

**DOI:** 10.3390/s19010208

**Published:** 2019-01-08

**Authors:** Christina Salchow-Hömmen, Leonie Callies, Daniel Laidig, Markus Valtin, Thomas Schauer, Thomas Seel

**Affiliations:** Control Systems Group, Technische Universität Berlin, Berlin 10587, Germany; leonie.callies@alumni.tu-berlin.de (L.C.); laidig@control.tu-berlin.de (D.L.); markus.valtin@campus.tu-berlin.de (M.V.); schauer@control.tu-berlin.de (T.S.); seel@control.tu-berlin.de (T.S.)

**Keywords:** inertial sensor, inertial measurement unit, real-time motion tracking, hand tracking, magnetic disturbances, dual quaternions, hand and finger kinematics, rehabilitation, functional electrical stimulation

## Abstract

Objective real-time assessment of hand motion is crucial in many clinical applications including technically-assisted physical rehabilitation of the upper extremity. We propose an inertial-sensor-based hand motion tracking system and a set of dual-quaternion-based methods for estimation of finger segment orientations and fingertip positions. The proposed system addresses the specific requirements of clinical applications in two ways: (1) In contrast to glove-based approaches, the proposed solution maintains the sense of touch. (2) In contrast to previous work, the proposed methods avoid the use of complex calibration procedures, which means that they are suitable for patients with severe motor impairment of the hand. To overcome the limited significance of validation in lab environments with homogeneous magnetic fields, we validate the proposed system using functional hand motions in the presence of severe magnetic disturbances as they appear in realistic clinical settings. We show that standard sensor fusion methods that rely on magnetometer readings may perform well in perfect laboratory environments but can lead to more than 15 cm root-mean-square error for the fingertip distances in realistic environments, while our advanced method yields root-mean-square errors below 2 cm for all performed motions.

## 1. Introduction

### 1.1. Motivation

Assistive technology for the recovery of patients who suffer from motor impairments due to an injury of the spinal cord (SCI) or due to a stroke is of increasing interest in an aging society. Functional electrical stimulation (FES) has proven to be a promising tool to help patients regain some mobility of the paralyzed limbs [1]. Many systems proposed in the literature employ an open-loop control of the neuro-muscular stimulation [2]. As the human body and the human hand in particular are complex, highly nonlinear, and time-variant systems, the same stimulation often leads to entirely different outcomes in different patients and in different rehabilitation sessions [3]. Closed-loop control of FES has the potential to improve the effectiveness and the usefulness of the therapy significantly. This has been demonstrated for gait support [4,5] as well as for upper limb motion support [6,7,8]. In the case of hand motor rehabilitation, a closed-loop approach requires real-time methods for accurate assessment of hand motion [9]. Furthermore, many FES-based systems utilize precise hand motion tracking for the identification of optimal stimulation points in electrode arrays on the forearm [10,11]. Beyond the feedback control and tuning of hand neuroprostheses, such methods would facilitate, for example, real-time biofeedback of hand motion and would yield valuable measurement information for robotic assistive devices [12].

Real-time hand motion tracking is a challenging task due to the large number of finger segments and degrees of freedom of the joints between them. Moreover, the field of rehabilitation imposes some specific requirements on the design of a hand motion tracking system. It needs to be portable to allow the application in various clinical environments or even in the context of supervised home rehabilitation. Complex calibration procedures must be avoided to make the system suitable for patients with severe motor impairment of the hand. For the same reason attaching the system to the hand must be easy and non-restrictive. For example, while gloves may be preferred in gaming applications, it is not a feasible solution for spastic hands. Furthermore, hygienic aspects have to be considered, especially if more than one patient uses the system.

Lastly, rehabilitation is typically performed in indoor environments and in the proximity of electronic devices and objects (e.g., tables, chairs, hand tools) containing ferromagnetic material such as steel. Therefore, the assumption of a homogeneous magnetic field is not fulfilled in a realistic rehabilitation setting, which is a problem when it comes to motion tracking with inertial measurement units (IMU). If an IMU passes by a ferromagnetic object, this induces a short-time magnetic disturbance that can be detected and treated by adjusting the sensor fusion weight. However, if the hand is placed near an electronic device or grasps a ferromagnetic object for a long period, the disturbance does not disappear quickly, and the magnetometer readings are useless during the entire time span. This severely impedes usability of most existing sensor fusion schemes [13,14]. A reliable system for hand motion tracking must measure the fingertip positions despite these challenges.

### 1.2. Previous Approaches to Hand Motion Tracking

Table 1 summarizes and compares available types of measurement systems for hand motion tracking in clinical applications. Many of the mentioned systems offer a high tracking accuracy for healthy hands and are optimized for the various applications in virtual and augmented reality. However, there are unsolved problems when it comes to the use in physical rehabilitation. Optical systems are subject to line-of-sight restrictions, i.e., they can only track finger segments that are inside the observation volume of stationarily mounted cameras and are not hidden by other segments or objects. Moreover, optical systems must either be set up by an expert (in the case of marker-based systems) or yield only limited accuracy (in the case of marker-free systems) [15].

Instrumented gloves have been proposed with different sensor technologies such as resistive, optical or inertial sensors [16,17]. Resistive-bend sensors and optical-fiber sensors are placed in gloves of varying material to cover the joints of interest [18]. This makes them prone to mechanical wear and it entails the need for a thorough calibration. Moreover, these sensors quantify joint angles and are not capable of measuring an absolute orientation of the finger segments (cf. Table 1). In contrast, inertial and magnetic sensors are placed on single finger segments and allow the estimation of velocities and orientations [19], but the calibration and magnetically disturbed environments can hamper practical application. In general, the usage of gloves has the distinct disadvantage of being difficult to put onto motor-impaired or even spastic hands of stroke and SCI patients. They must be customized to individual hand sizes, lead to a reduced sense of touch, and cleaning plus disinfecting is challenging.

These shortcomings are well reflected in the small number of existing studies that propose systems for closed-loop FES based on real-time sensing of hand motions. Soska et al. [2] introduce a method to control the joint angles of the affected hand by an iterative learning controller but present only simulative results without proposing solutions for hand motion tracking. Westerveld et al. [21] used a closed-loop setup in which they selectively activate and control individual finger movements. They employed a marker-based optical motion capture system to track finger flexion angles as well as thumb abduction and extension angles. A proportional and a model-predictive controller were designed and tested in able-bodied volunteers for grasping, holding and releasing two different objects. Kim et al. [22] used a 2D gyroscope on the back of the hand to detect the direction of the hand motion and a ring-type accelerometer on each finger to measure finger motion. The device is capable of recognizing simple hand poses and identifying finger-clicking, which means the system can be used as a wearable computer mouse. It is, however, not capable of estimating the finger segment orientations and fingertip positions, which are needed to track and quantify the motion of the hand and its interaction with objects.

Kortier et al. [19] further pursue the idea of using gyroscope and accelerometer data for the tracking of hand motions with a data glove. They apply IMUs consisting of a 3D gyroscope and a 3D accelerometer to all finger segments and three locations on the back of the hand. In addition, 3D magnetometers are placed at the fingertips and on the back of the hand. Multiple extended Kalman filters are used to fuse the sensor readings and biomechanical constraints to estimate the orientation of every sensor. Each measurement is preceded by a calibration protocol that consists of prescribed poses and precisely defined finger motions, which are used to determine the axes of rotation of all joints. The authors compare their estimation to an optical system in trials with pinching movements of thumb and index finger [19,20]. All results are obtained in a laboratory environment under the assumption of a perfectly homogeneous magnetic field. Such conditions are rarely found in clinical practice, and a large number of neurological patients will be unable to perform the required precise calibration poses and motions properly.

Connolly et al. [23] present an IMU-based glove system that aims at measuring finger and thumb joint movements accurately in patients with rheumatoid arthritis, who are capable of donning a glove. The system uses inertial sensors connected via stretchable substrate material to calculate joint angles and angular velocities. The inertial sensor fusion is based on magnetometers, which will yield false measurements in realistic therapy environments. The motion estimation method requires an initial calibration pose. The description of the specific algorithm is very brief, contains no equations, and indicates that joint angle calculation is based on measured gravitational accelerations, which implies that the method becomes highly inaccurate if the joint axis is close to vertical. Under laboratory conditions, the system exhibited root-mean-square errors (RMSE) of 6° when placed on wooden blocks cut to specific angles.

Choi et al. [24] propose a wireless IMU-based glove system with modular design and a new orientation estimation algorithm. The algorithm relies on undisturbed magnetometer data and requires a time-consuming calibration procedure. Their evaluation lacks real-time measurements in realistic conditions and numeric results with human hands. Recently, Lin et al. [25] presented a similar design. They report a mean error in joint angles under ±3° for a 15 min measurement of extension and flexion. However, they evaluated the system on a customized measurement platform, allowing only extension and flexion of finger joints, in a laboratory environment under the assumptions of a perfectly homogeneous magnetic field and with stable sensor biases. It remains unclear how the system performs on the human hand, where many joints have more than one degree of freedom.

In summary, there exist very few previous contributions that propose solutions for real-time hand motion tracking. With respect to the requirements of motor rehabilitation in stroke and SCI patients, further developments are needed. More precisely, a system is required that is easy to put onto spastic hands, does not rely on magnetometers, is not subject to line-of-sight restrictions, and requires only a minimum of calibration effort. If such a system can be developed, its accuracy should be evaluated for functional motions like pinching and grasping objects in a realistic environment with substantial and permanent magnetic field disturbances.

### 1.3. The Proposed Approach

**Figure 1:**
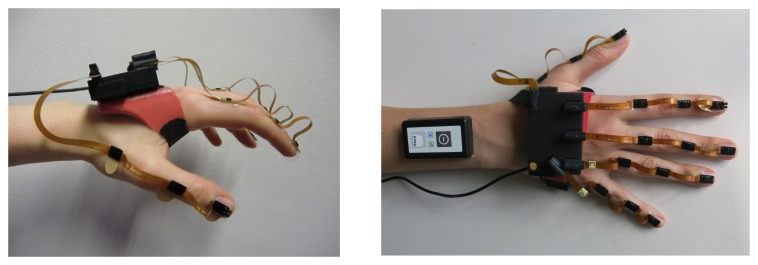
IMU-based modular hand sensor system for real-time motion tracking of the fingertip positions. The system consists of a base unit on the hand back, a wireless IMU on the forearm, and up to five sensor strips, each equipped with three IMUs.

In the current contribution, we propose a portable IMU-based sensor system for real-time tracking of fingertip positions that can, for example, be used in a feedback-controlled hand neuroprosthesis. The system is composed of a base unit, which is placed on the back of the hand, and five sensor strips, which are placed on the segments of the fingers, as depicted in Figure 1. By adhesive attachment, we avoid the aforementioned disadvantages of gloves. The proposed motion estimation method is based on the mathematical framework of dual quaternions and aims at avoiding complex calibration motions and at meeting the requirements of clinical rehabilitation practice. We propose a set of algorithms using a biomechanical model of the hand that includes all five fingers and captures the 23 main rotational degrees of freedom (DoF) of the hand and finger joints. Besides a method that uses the full IMU raw data, we also propose a method that refrains from using magnetometer readings to assure robust motion tracking in the presence of non-ideal magnetic fields. Both methods are compared to a baseline approach by experimental trials in an optical motion capture lab and in a more realistic environment with magnetic disturbances. For evaluation and comparison, we focus on the accuracy of fingertip positions since these are crucial for functional motions of the hand such as pinching or grasping.

The remainder of the article is organized as follows. We introduce the sensor system and the necessary biomechanical foundations in Section 2. We continue with an introduction to dual quaternions and the description of the estimation algorithm. The experimental validation procedure is described in Section 3. The experimental results are presented in Section 4 and are discussed in Section 5.

## 2. Materials and Methods

### 2.1. Hand Sensor System Hardware

The novel IMU-based hand sensor system shown in Figure 1 was recently developed as part of a feedback-controlled neuroprosthesis [26,27,28]. The hardware setup was inspired by Kortier et al. [19]. It consists of a mandatory base unit that is placed on the back of the hand, up to five sensor strips that can be fixed to the fingers, and an optional wireless inertial sensor for the forearm to track the wrist angles if desired. The system is compact and portable as well as modular in the sense that sensor strips can be removed and replaced arbitrarily. These characteristics increase the flexibility in different therapy settings and for multiple hand sizes making the system more practical and easier to maintain.

The base unit is a custom printed circuit board placed in a 3D printed shell (6.1×4.0×1.1 cm). It includes a 9D inertial sensor (MPU9259, InvenSense Inc., San Jose, CA, USA; footprint 3×3 mm) and five connectors for the sensor strips. A USB connection to the computer facilitates power supply and data transfer.

To capture hand motions in high detail, the sensor system measures the translational and rotational motion of each finger segment and the hand back. Consequently, each sensor strip connects three 9D inertial sensors (MPU9259) through a 19 cm long flexible printed circuit board. One sensor is attached to each of the three segments of the finger, as depicted in Figure 1. The strips at the thumb, index, and middle finger have additional connectors for optional pressure sensors at the finger end segment, which can be taped to the fingertip to measure grasp strength.

Focusing on stroke and SCI patients, we refrained from embedding the system into a glove and instead attached individual sensor strips adhesively to the finger segments using skin-friendly tape. In this way, the user has full sense of touch, which is important when relearning to manipulate objects. A silicon fixture was designed that attaches the base unit of the system to the hand back. The mounting of the hand sensor systems by another person takes approximately 2 min. While the suggested setup may not be superior in sports or virtual reality applications, it is particularly suitable for clinical use with paralyzed hands, as it can be mounted on closed hands (e.g., if a voluntary extension is impeded due to high muscle tone). The complete system measures a total weight of 50 g (25 g base unit + 15 g silicon fixture 2 g per sensor strip), which equals approximately 10% of the average human hand [29].

**Figure 2:**
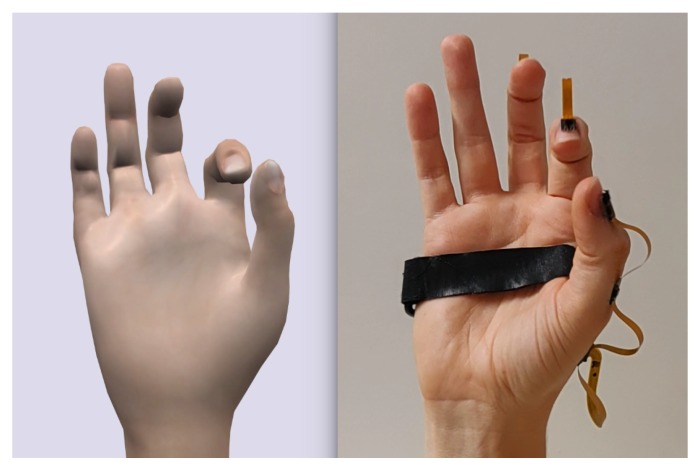
3D real-time visualization of the measured hand posture.

Data transfer between the sensor strips and the unit is provided by a serial peripheral interface bus. Sensor data can be sampled at frequencies of up to 1 kHz for accelerometers and gyroscopes, whereas the magnetometers are limited to 100 Hz. The sensor for the forearm (HASOMED GmbH, Magdeburg, Germany) is independent of the base unit and sends its data via Bluetooth (3.0 HDR; high data rate) directly to the computer at the same frequency and with a latency of approximately 10 ms. The forearm sensor is necessary for tracking wrist joint angles in various arm positions. Processing of the raw IMU data is done in Matlab and Simulink (MathWorks, Natick, MA, USA). To monitor the hand motion in real time, an animated 3D visualization has been developed using Matlab/Simulink for real-time data processing and BabylonJS as a 3D engine. An example of the visualization is shown in Figure 2.

Calibration of all sensors was performed using a custom-built calibration robot to automate the process and to ensure a consistent calibration quality [30]. Besides the bias, we also determined a linear scaling correction factor, a rotation matrix that ensures the orthogonality of the sensor axes, and a second rotation matrix, which assures that all three sensor types (accelerometer, magnetometer, gyroscope) have the same orientation. This sensor calibration was performed once and was not repeated prior to any of the measurement trial described in Section 3.

### 2.2. Biomechanical Hand Model

Inertial sensors can be used to determine the orientation of body segments in a global coordinate frame via sensor fusion [14]. However, our goal is to track fingertip positions. Therefore, we need a biomechanical model of the hand that describes the relations between segment orientations and positions [31]. Various models are found in the literature, and they differ significantly in their degree of complexity. Cobos et al. [32,33] give an overview of literature that introduces models with 26, 24, 23 and 20 degrees of freedom for the fingers. For our purposes, we model the hand as a kinematic chain with 21 rotational DoF for the finger joints and two DoF for the wrist (in total 23 DoF).

#### 2.2.1. Anatomy of the Hand

**Figure 3:**
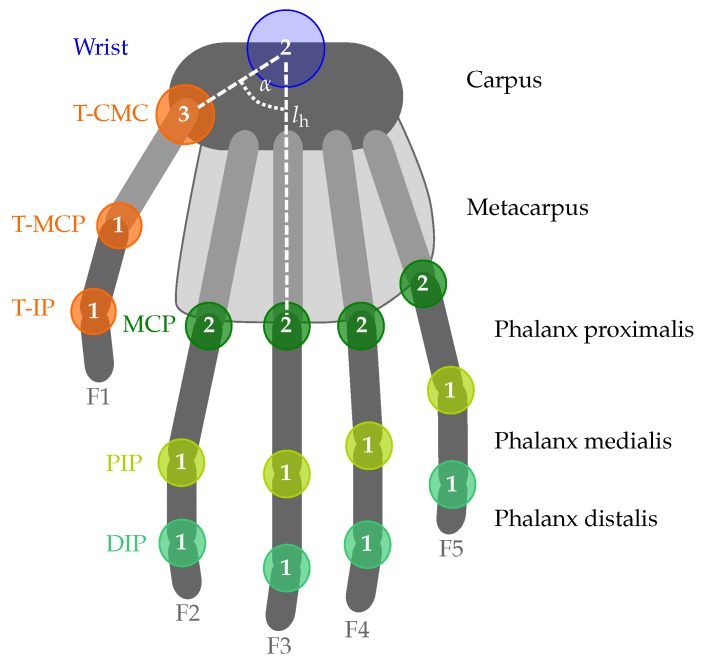
Modeled bones and joints of the human hand. The fingers are numbered from F1 (thumb) to F5 (little finger). The modeled joints are illustrated as colored circles with numbers indicating the considered degrees of freedom of the joint. The metacarpals (light gray) of F2–F5 form the palm, which is treated as flat and rigid. The white dotted line from the wrist to the MCP joint of the middle finger marks the length of the palm $l_{h}$, which needs to be measured for the model. The remaining joint center positions of MCP and T-CMC are deviated via constant ratios and angles, as exemplarily shown for the thumb with angle α=58°.

In preparation of the subsequent explanations, a few terms, designations and model assumptions are introduced in the following. The five fingers will be denoted by F1 to F5, where F1 indicates the thumb and F5 the little (most ulnar) finger, as illustrated in Figure 3. Fingers F2–F5 consist of three phalanges (segments). Starting from the fingertip, the phalanges are called *distal*, *middle* and *proximal phalanx*. The phalanges are connected by two hinge joints with one DoF. These joints are named *distal* and *proximal interphalangeal* joints (DIP and PIP) respectively (see Figure 3). F2 to F5 are connected to the palm by saddle joints with two DoF named *metacarpal-phalangeal* joints (MCP; cf. Figure 3). The palm itself consists of the *metacarpals* that are connected to the forearm through eight carpal bones. These carpals form the wrist and allow for complex movements such as arching the palm [34,35]. However, our model simplifies the wrist by replacing it with a saddle joint. This implies that the palm of the hand is assumed to be flat and rigid. The position of the MCP joints can be obtained by pure and constant translation along the palm.

The thumb consists of a metacarpal bone, a proximal, and a distal phalanx (see Figure 3). The latter are connected through a hinge joint (*thumb-interphalangeal* joint, T-IP), likewise the proximal phalanx and the metacarpal of the thumb (*thumb-metacarpal-phalangeal* joint, T-MCP). The thumb’s metacarpal bone is linked to the wrist by a spheroidal ball joint that has three degrees of freedom (*thumb-carpo-metacarpal* joint, T-CMC). This assumption guarantees that the opposition of the thumb to the palm can be modeled.

**Figure 4:**
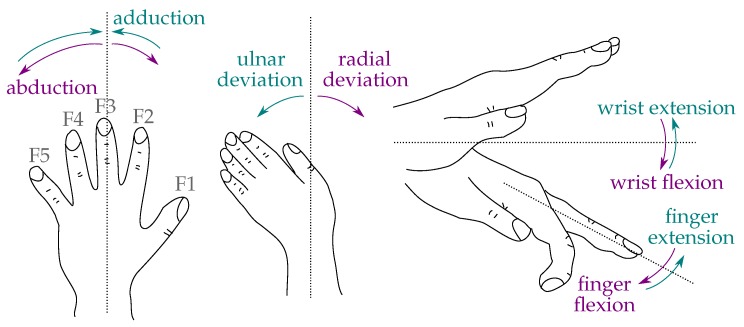
Definition of hand and finger movements, illustrated for the left hand. Ulnar and radial deviation are also known as ulnar/radial abduction or wrist adduction. Finger abduction in the MCP joints describes the movement away from the center of the extremity, whereas finger adduction refers to the movement towards the center of the extremity.

Movements of the hand and fingers can be classified as *abduction/adduction* (fingers) and *ulnar/radial deviations* (wrist) for motions in the plane of the hand and *flexion/extension* for motions that are perpendicular to this plane. Those movements are illustrated in Figure 4.

#### 2.2.2. Definition of Local Coordinate Systems

The International Society of Biomechanics (ISB) has proposed a set of coordinate systems to be used when reporting kinematic data of the human body [36]. For hand and forearm, the authors introduce a coordinate system with an *x*-axis pointing in dorsal (left hand) or palmar (right hand) direction. The *y*-axis coincides with the longitudinal axis of the bone and is directed distally for the left hand and proximally for the right hand. The *z*-axis is added to form a right-handed coordinate system.

This is only one possible definition of coordinate systems. Goislard de Monsabert et al. [37] have compared and tested two different approaches. They call the second concept *functional axes*. The *z*-axis of this approach is the actual axis of flexion and extension in the finger joints which does not necessarily coincide with the *z*-axis of the ISB convention. It is used, for instance, by Kortier et al. [19]. This axis is not constant during flexion of the finger [37,38]. Goislard de Monsabert et al. [37] conclude that the functional axes facilitate the interpretation of the results, but that the differences in the angles about the two varying *z*-axes are minor, namely always less than 7° for the same movement. It is more complicated to identify and use functional axes because it requires complex calibration movements. Therefore, the authors do not give a strong recommendation. Consequently, we choose to use coordinate systems according to the ISB recommendations.

**Figure 5:**
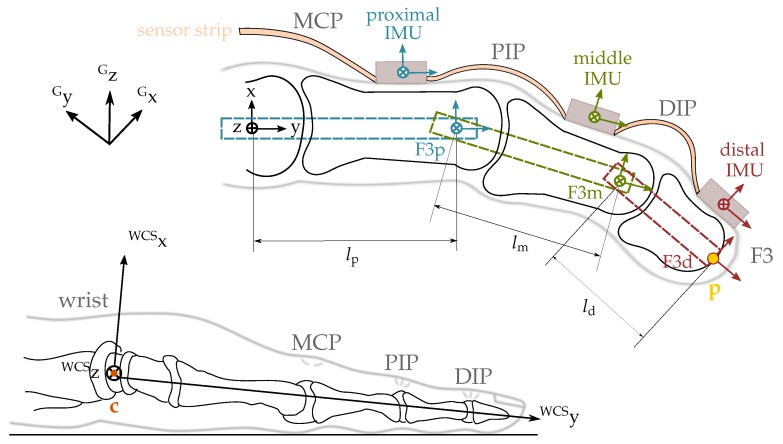
Top: model terms for the exemplary finger F3 of a left hand and location of coordinate frames: IMU frames as well as bone frames (both no index) located in the centers of rotation and the inertial global coordinate frame (index G). The IMU and bone frames are assumed to differ only by a constant translational offset. $l_{p}$, $l_{m}$, and $l_{d}$ denote the functional lengths of the proximal, middle, and distal phalanges. The point p marks the fingertip position of interest. Bottom: wrist coordinate system (WCS) located in the center c of rotation of the wrist for the left hand.

In the biomechanical model of the hand, we associate each bone with a coordinate frame that has the same orientation as the rigidly connected IMU but lies on the longitudinal axis of the bone at the distal center of rotation (or fingertip in the case of the distal phalanges) and has axes that are named according to the ISB recommendations. These coordinate systems are illustrated in Figure 5 for the example of the middle finger F3. In the following, they will be denoted by the segment index j∈S= {forearm, hand, F1p, F1m, F1d, F2p, F2m, F2d, F3p, F3m, F3d, F4p, F4m, F4d, F5p, F5m, F5d}.

The coordinate frame in which we determine the fingertip positions has its origin in the center of rotation of the wrist and has a constant, fixed orientation with respect to the forearm, as depicted in Figure 5 (bottom). It is denoted wrist coordinate system (WCS). The *y*-axis coincides with the middle finger and points distally when the arm and hand lie flat on a horizontal surface and the abduction angle of the wrist joint is zero. This pose is defined as the neutral pose. It implies that the *y*-axis is slightly tilted downward and the *x*-axis deviates by a few degrees from the vertical axis. Therefore, the *y*-axis is not exactly aligned with the longitudinal axis of the forearm.

#### 2.2.3. Lengths of the Phalanges

The kinematic model needs to be completed by the lengths of the bones. Several studies on relative lengths of the phalanges have been conducted [39,40,41,42]. The use of relative lengths has the advantage that only a small number of characteristic quantities must be measured for accurate tracking of an individual hand rather than measuring each phalanx individually. This reduces the required time and lowers the risk of additional measurement errors. The studies mentioned above distinguish between ratios of bone lengths and ratios of functional lengths. The latter describe distances between the centers of rotation of the bones and are the lengths that are of interest in our case. The results presented in [39,40,41,42] are summarized in Table 2, they agree very well with each other. Therefore, we use these ratios to calculate functional segment length from hand length measurement in the following.

Buryanov et al. [42] have also examined the amount of soft tissue at the fingertip. The average thicknesses are provided in Table 3. These are subtracted from the measured lengths of the fingers before the ratios are applied to determine the segment lengths in our hand model.

Likewise, the length of the palm needs to be measured from the wrist to the MCP joint of F3. Constant angles and ratios are used to determine the position of T-CMC joint of the thumb and the MCP joints of the remaining fingers respectively, as illustrated in Figure 3.

### 2.3. Introduction to Quaternions and Dual Quaternions

The estimation of segment orientations and fingertip positions is based on the concept of quaternions, which enable an elegant and gimbal-lock-free description of rotations in $R^{3}$. Beyond that, the extension to dual quaternions facilitates a description of both rotations and translations in $R^{3}$ that exhibits no singularities and is more compact than other representations [43]. Our implementations are based on the Matlab toolbox for dual quaternions developed by Leclercq et al. [44]. In the following, we give a brief introduction to the mathematical concept of dual quaternions. For a detailed introduction, please refer to Leclercq et al. [44] and references therein.

#### 2.3.1. Definitions

A *quaternion* is defined as

(1) $$q=q_{0}+q_{1}i+q_{2}j+q_{3}k$$

with the three imaginary units i,j,k, which satisfy the Hamilton identity [45]

(2) $$i^{2}=j^{2}=k^{2}=ijk=-1.$$

The set of all quaternions is denoted by H. The imaginary units can be interpreted as an orthonormal basis of $R^{3}$ with $i={\left[1,0,0\right]}^{T}$, $j={\left[0,1,0\right]}^{T}$ and $k={\left[0,0,1\right]}^{T}$. We can thus write the quaternion as the sum of a scalar $q_{0}$ and a vector part

(3) $$\begin{array}{c} q=q_{0}+\left[\begin{array}{c} q_{1}\\ q_{2}\\ q_{3} \end{array}\right] \end{array}$$

and use common vector operations on the vector part. It follows from Equation (2) that multiplication of two quaternions $\hat{q}$ and q yields the quaternion

(4) $$\hat{q}\;q={\hat{q}}_{0}q_{0}-\left[\begin{array}{c} {\hat{q}}_{1}\\ {\hat{q}}_{2}\\ {\hat{q}}_{3} \end{array}\right]\;\cdot \;\left[\begin{array}{c} q_{1}\\ q_{2}\\ q_{3} \end{array}\right]+{\hat{q}}_{0}\left[\begin{array}{c} q_{1}\\ q_{2}\\ q_{3} \end{array}\right]+q_{0}\left[\begin{array}{c} {\hat{q}}_{1}\\ {\hat{q}}_{2}\\ {\hat{q}}_{3} \end{array}\right]+\left[\begin{array}{c} {\hat{q}}_{1}\\ {\hat{q}}_{2}\\ {\hat{q}}_{3} \end{array}\right]\times \left[\begin{array}{c} q_{1}\\ q_{2}\\ q_{3} \end{array}\right],$$

where · denotes the scalar product and × denotes the cross product. The *conjugate* of a quaternion is defined as

(5) $$q^{*}=q_{0}-q_{1}i-q_{2}j-q_{3}k,$$

and the *quaternion norm* of q is

(6) $$∥\;q∥=\sqrt{\;q\;q^{*}}=\sqrt{q_{0}^{2}+q_{1}^{2}+q_{2}^{2}+q_{3}^{2}}.$$

A quaternion with ∥q∥=1 is called *unit quaternion*. As the quaternion norm is multiplicative, the multiplication of two unit quaternions always yields a unit quaternion.

A *dual number* is defined to be z=r+ϵd, where r,d∈R. ϵ is called the *dual unit* and fulfills the condition $\epsilon^{2}=0$ with ϵ≠0 [44]. In analogy to complex numbers, *r* is called the real part and *d* the dual part of *z*.

Combining the two aforementioned concepts, a *dual quaternion* is defined as

(7) $$Q=q_{r}+\epsilon \;q_{d}$$

with $q_{r},\;q_{d}\in H$ [46]. The set of dual quaternions is called D.

To distinguish dual from regular quaternions (which are denoted by lowercase letters), dual quaternions will be denoted by uppercase letters. Multiplication of two dual quaternions $Q_{A}$ and $Q_{B}$ can be expressed by quaternion multiplications of the real and dual parts and results in

(8) $$\begin{array}{ccc} Q_{A}\;Q_{B} & = & \left(q_{\mathrm{Ar}}+\epsilon \;q_{\mathrm{Ad}}\right)\left(q_{\mathrm{Br}}+\epsilon \;q_{\mathrm{Bd}}\right)\\ \; & = & q_{\mathrm{Ar}}\;q_{\mathrm{Br}}+\epsilon \left(q_{\mathrm{Ar}}\;q_{\mathrm{Bd}}+q_{\mathrm{Br}}\;q_{\mathrm{Ad}}\right). \end{array}$$

The conjugate of a dual quaternion can be defined in multiple ways depending on the application. For the calculation of rigid body transformations, the following definition is useful. The dual quaternion

(9) $$Q^{*}=q_{r}^{*}-\epsilon \;q_{d}^{*}$$

is called the *conjugate* of Q, where $q_{r}^{*}$ and $q_{d}^{*}$ are quaternion conjugates according to Equation (5).

A dual quaternion is called *unit* dual quaternion if

(10) $$\begin{array}{ccc} \;∥\;q_{r}∥ & = & 1, \end{array}$$

(11) $$\begin{array}{ccc} q_{r}\;q_{d}^{*}+\;q_{d}\;q_{r}^{*} & = & 0. \end{array}$$

Furthermore, the set of dual quaternions with a real part equal to one and a dual part with zero scalar part is denoted by $D_{0}$. Both the set $D_{0}$ and the set of unit dual quaternions are necessary to describe 3D kinematics with dual quaternions.

#### 2.3.2. Describing Rotations and Translations with Dual Quaternions

Describing rotations and translations of objects in $R^{3}$ by dual quaternions requires a relationship between the vectors and dual quaternions. Following Leclercq et al. [44], we assign a unique dual quaternion $Q\in D_{0}$ to each vector $v\in R^{3}$ by choosing the vector as the vector part of the dual part of Q and setting the real part of Q to one.

**Definition** **1.** *Let $v\in R^{3}$. We define the bijective operator*

(12) $$D:\;R^{3}\to D_{0},\;v\mapsto D\left(v\right)=\left(1+0\right)+\epsilon \left(0+v\right)$$

*that yields the dual quaternion equivalent D(v) for any vector v.*

Using this relationship, we define the dual-quaternion operator that maps a given vector onto another (rotated and translated) vector.

**Definition** **2.** *Let $v\in R^{3}$ and let Q be a unit dual quaternion. The operator*

(13) $$L\left(\;\;\cdot \;\;,\;Q\right):R^{3}\to R^{3},\;v\mapsto L\left(v,\;Q\right)=D^{-1}\left(\;Q\;D\left(v\right)\;Q^{*}\;\right)$$

*is called the* dual-quaternion operator.

Note that v is mapped to its dual-quaternion equivalent, then multiplied from both sides by Q and its conjugate, and then mapped back into $R^{3}$. The geometric interpretation of this operator is given by the following theorem.

**Theorem** **1.** *According to [44], let $r,t\in R^{3}$ be unit vectors, θ∈[−π,π], l∈R and $v\in R^{3}$. Consider the dual quaternions*

(14) $$\begin{array}{ccc} Q_{r} & = & \cos\left(\frac{\theta }{2}\right)+r\sin\left(\frac{\theta }{2}\right)+\epsilon \;\cdot \;0\;=:\;q_{\mathrm{rr}}+\epsilon \;q_{\mathrm{rd}}, \end{array}$$

(15) $$\begin{array}{ccc} Q_{t} & = & 1+\epsilon \frac{l}{2}\left(0+t\right)\;=:\;q_{\mathrm{tr}}+\epsilon \;q_{\mathrm{td}}. \end{array}$$

*Then, $Q_{r}$ and $Q_{t}$ are unit dual quaternions, and the dual-quaternion operation $L(v,\;Q_{r})$ describes a rotation of v by θ around the rotation axis r, and $L(v,\;Q_{t})$ describes a translation of v of length l along t.*

Having described rotations and translations in the same mathematical framework, we can now easily describe combinations of rotation and translations by dual-quaternion multiplication.

(16) $$\begin{array}{cc} Q & =Q_{r}\;Q_{t}=q_{\mathrm{rr}}\;q_{\mathrm{tr}}+\epsilon \left(\;q_{\mathrm{rr}}\;q_{\mathrm{td}}+\;q_{\mathrm{rd}}\;q_{\mathrm{tr}}\right)\\ \; & =\cos\left(\frac{\theta }{2}\right)+r\sin\left(\frac{\theta }{2}\right)+\epsilon \frac{l}{2}\left[-\sin\left(\frac{\theta }{2}\right)r\;\cdot \;t+\cos\left(\frac{\theta }{2}\right)t+\sin\left(\frac{\theta }{2}\right)r\times t\right]. \end{array}$$

In the sequel, we will often have the special case of a rotation followed by a translation that is orthogonal to the rotation axis. In that case, the scalar product in the above equation, i.e., the scalar part of the dual part, becomes zero.

Note that any combination of rotations and translations can be described by a single unit dual quaternion. The two unity conditions (10) and (11) reduce the number of DoF from eight (general dual quaternion) to six (unit dual quaternion), which corresponds to the DoF of rotation and translation a rigid body in 3D.

Concatenation of rotations and translations is achieved by simple multiplication of the respective dual quaternions. However, attention must be paid to the order of multiplication and to whether rotations are extrinsic or intrinsic. Extrinsic rotations are rotations about (global) axes that are fixed in space, whereas intrinsic rotations are rotations about (local) body-fixed axes. Both lead to different order of multiplication of the quaternions. In the following, we consider concatenation of intrinsic rotations to describe hand and finger kinematics. We introduce the following notation: left subscripts denote the coordinate system in which the quaternion or vector is given, and left superscripts denote the object whose orientation is considered. Hence, ${}_{G}^{S}Q$ denotes the orientation of segment S in the global coordinate system denoted by index G.

For illustration of the concatenation, consider two segments that are linked by a joint. The orientation and position of the first segment is given by ${}_{G}^{S1}Q$, while the orientation and position of the second segment is given relative to the first by ${}_{S1}^{S2}Q$. The second segment’s orientation and position in the global frame is then given by ${}_{G}^{S2}Q={}_{G}^{S1}Q\;{}_{S1}^{S2}Q$.

### 2.4. The Hand Sensor System Algorithm

**Figure 6:**
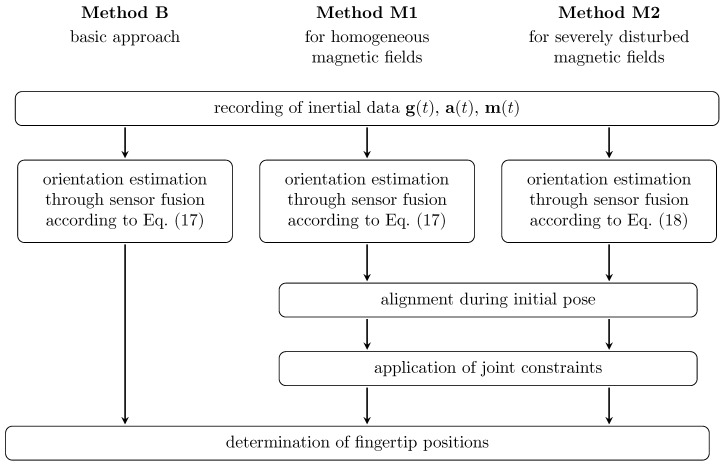
Summary of three proposed fingertip position estimation methods: the baseline method (B), and two advanced methods (M1 and M2). M1 and M2 exploit joint constraints to compensate errors in the attachment and the orientation estimation. In contrast to M1, M2 is completely magnetometer-free and thus suitable for environments with severely disturbed magnetic fields.

The segment orientations and fingertip positions shall be determined from the inertial measurements of the IMUs attached to the fingers and hand back. We present three approaches for the solution of this task: a baseline approach (method B) and two more advanced algorithms (methods M1 and M2), which exploit joint constraints to compensate errors in the attachment and the orientation estimation. In contrast to M1, the method M2 does not use measurements of the local magnetic field when estimating the segment orientations. Figure 6 summarizes the sub-steps of all three approaches, which we discuss in detail in the following.

#### 2.4.1. Data Recording and Sensor Fusion (B, M1, M2)

Inertial data is recorded at a frequency of f=100Hz and processed by a recently developed sensor fusion algorithm [14]. The algorithm performs strap-down integration of the angular rates g(t), with *t* being the time, to predict the sensor orientation and uses the magnetometer readings m(t) and accelerometer readings a(t) to compensate for integration drift. The latter is done in a way that assures that magnetometer-based corrections affect only the heading of the orientation estimate but not the inclination, which overcomes a drawback of most previous algorithms. Therefore, it facilitates estimation of the full orientation (inclination and heading) while limiting the influence of magnetic field disturbances to a minimum. Details are described in Seel et al. [14]. For the sake of compactness, we describe the sensor fusion algorithm by the recursive function F(·), which yields the dual orientation quaternion for a set of given sensor readings

(17) $$\begin{array}{c} {}_{G}^{j}Q_{r}\left(t\right)=F({}_{G}^{j}Q_{r}\left(t-t_{s}\right),g^{j}\left(t\right),a^{j}\left(t\right),m^{j}\left(t\right)), \end{array}$$

where j∈S denotes the segment to which the IMU is attached (cf. Section 2.2) and $t_{s}$ denotes the sampling time. As mentioned earlier, we want to propose methods that work in realistic settings with severe long-time or even permanent magnetic disturbances. In such situations, it is an inevitable consequence that any magnetometer-based correction of the strap-down integration deteriorates the heading of the orientation estimate. To assure accurate motion tracking even in severely disturbed magnetic fields, we propose method M2 that completely avoids the use of magnetometer readings. Only accelerometer-based corrections are performed [14], and absolute heading information is instead obtained from an initial pose. In analogy to the above, the magnetometer-free sensor fusion is described by the recursive function $\tilde{F}\left(\;\cdot \;\right)$:

(18) $$\begin{array}{c} {}_{G}^{j}Q_{r}\left(t\right)=\tilde{F}\left(\;{}_{G}^{j}Q_{r}\left(t-t_{s}\right),g^{j}\left(t\right),a^{j}\left(t\right)\right), \end{array}$$

where j∈S denotes the segment to which the IMU is attached.

#### 2.4.2. Initial Pose Alignment (M1, M2)

If the local magnetic field is not perfectly homogeneous, the relative heading between inertial sensors cannot be determined from the magnetometer readings. To overcome this limitation, we use an initial pose period of at least three seconds during which fingers F2 to F5 are straight, and the straight thumb is either abducted at a known angle or aligned with the other fingers. Any relative heading measured by the magnetometers during that pose is then removed, and the initial-pose-aligned orientation quaternions ${}_{G}^{j}Q_{r,\mathrm{ipa}}\left(t\right)$ for each segment j∈S are obtained. Moreover, the initial-pose period is used to estimate and compensate for the gyroscope bias, which improves the accuracy of the sensor fusion.

#### 2.4.3. Application of Joint Constraints (M1, M2)

In each time step *t*, we calculate the relative orientation

(19) $$\begin{array}{cc} {}_{b}^{a}Q_{r,\mathrm{ipa}}\left(t\right) & ={}_{G}^{b}Q_{r,\mathrm{ipa}}^{*}\left(t\right)\;\;\;{}_{G}^{a}Q_{r,\mathrm{ipa}}\left(t\right),\\ \; & =q_{0}+q_{1}i+q_{2}j+q_{3}k+\epsilon 0, \end{array}$$

between two adjacent sensors a,b ∈S and convert the relative quaternion ${}_{b}^{a}Q_{r,\mathrm{ipa}}\left(t\right)$ to intrinsic zxy-Euler angles [27,36] using

(20) $$\begin{array}{ccc} \phi_{z} & = & atan2\left(2\left(q_{0}q_{3}-q_{1}q_{2}\right),q_{0}^{2}-q_{1}^{2}+q_{2}^{2}-q_{3}^{2}\right), \end{array}$$

(21) $$\begin{array}{ccc} \phi_{x} & = & \arcsin(2\left(q_{0}q_{1}+q_{2}q_{3}\right)),\; \end{array}$$

(22) $$\begin{array}{ccc} \phi_{y} & = & atan2\left(2\left(q_{0}q_{2}-q_{1}q_{3}\right),q_{0}^{2}-q_{1}^{2}-q_{2}^{2}+q_{3}^{2}\right). \end{array}$$

We then discard any non-physiological rotation around axes that are not any of the degrees of freedom of the respective biological joint model. For example, in PIP joints, the local *z*-axis represents a hinge joint axis, and thus $\phi_{x}$ and $\phi_{y}$ are set to zero. Moreover, we restrict the angles around physiological axes to an anatomically permissible interval (DIP: F∈[−20°,100°]; PIP: F∈[−20°,120°]; T-IP: F∈[−20°,100°]). The intervals were extracted from literature and added with a tolerance [47]. The corrected Euler angles are then transformed back into quaternion space, which yields the modified relative orientation ${}_{b}^{a}{\tilde{Q}}_{r,\mathrm{ipa}}\left(t\right)$.

#### 2.4.4. Determination of the Fingertip Positions (B, M1, M2)

In the final step, the relative orientations of the sensors need to be combined with the translations between the joints. Recall that the coordinate system of each distal phalanx is defined to lie at the segment’s distal end, while the coordinate systems of the other segments are defined to lie in the distal joint center of those segments, as depicted in Figure 5. Consequently, the location and orientation of the F3 fingertip with respect to the F3m coordinate system are given by the relative quaternion

(23) $${}_{F3m}^{F3d}Q\left(t\right)={}_{F3m}^{F3d}{\tilde{Q}}_{r,\mathrm{ipa}}\left(t\right)\;\;\;{}_{F3m}^{F3d}Q_{t}.$$

For the example of the left hand, where the constant dual quaternion ${}_{F3m}^{F3d}Q_{t}=1+\epsilon \frac{l_{d}}{2}\left(0+{\left[0,1,0\right]}^{T}\right)$ describes the translational offset in direction of the *y*-axis (bone-centered axis) of the F3d segment, and $l_{d}$ was determined in Section 2.2. Likewise, we find that

(24) $${}_{F3p}^{F3m}Q\left(t\right)={}_{F3p}^{F3m}{\tilde{Q}}_{r,\mathrm{ipa}}\left(t\right)\;\;\;{}_{F3p}^{F3m}Q_{t}$$

connects the F3m segment with the F3p segment. The connections between the other segments and the segments of all other fingers are made analogously. Eventually, a compact expression of the entire kinematic chain of each finger is obtained by the dual quaternion

(25) $${}_{\mathrm{WCS}}^{Fid}Q\left(t\right)={}_{\mathrm{WCS}}^{\mathrm{hand}}Q\left(t\right)\;\;\;{}_{\mathrm{hand}}^{Fip}Q\left(t\right)\;\;\;{}_{Fip}^{Fim}Q\left(t\right)\;\;\;{}_{Fim}^{Fid}Q\left(t\right)$$

for $i\in \left\{2,3,4,5\right\}$ and by

(26) $${}_{\mathrm{WCS}}^{F1d}Q\left(t\right)={}_{\mathrm{WCS}}^{F1p}Q\left(t\right)\;\;\;{}_{F1p}^{F1m}Q\left(t\right)\;\;\;{}_{F1m}^{F1p}Q\left(t\right)\;\;\;{}_{F1m}^{F1d}Q\left(t\right)$$

for the thumb (F1).

Since the fingertips are the origins of the distal segments’ coordinate systems, the IMU-based fingertip positions p are calculated by applying the dual-quaternion operator to the zero vector

(27) $${}_{\mathrm{WCS}}^{Fid}p_{\mathrm{IMU}}\left(t\right)=L\left(0,\;{}_{\mathrm{WCS}}^{Fid}Q\left(t\right)\right)$$

for all five fingers *i*. The left subscript of p indicates the reference coordinate frame, while the right subscript indicates that the position was calculated from inertial measurements, denoted as IMU.

## 3. Experimental Validation

The accuracy of the proposed real-time hand motion tracking system is evaluated in experimental trials with four able-bodied volunteers (three female, one male, age 29.5 ± 2.65). The experiments have been approved by the local ethical committee (Berlin Chamber of Physicians, Eth-25/15). Written informed consent was obtained from each participant before the session.

Experiments are performed in two different settings. First, we conduct experiments with one participant (#1) in an idealistic laboratory environment without magnetic disturbances, as it is common in previous literature. We compare the proposed hand motion tracking system with a marker-based optical motion capture system to the facilitate comparison of our results to earlier publications with similar setups. To avoid marker occlusion and marker swapping, we perform isolated movements of single fingers. For the sake of a more realistic assessment, we consider a second setting with experiments in all four participants (#1–#4) involving functional hand postures and motions in environments with disturbed magnetic fields. In these experiments, we use knowledge about contact or distance between fingertips and objects to assess the accuracy of the proposed methods. In both settings, only the thumb (F1) and the index and middle fingers (F2, F3) are considered because they contribute most to functional motions such as grasping, pinching or pointing.

### 3.1. Idealistic Setting with Optical Reference System (Setting 1)

#### 3.1.1. Setup

**Figure 7:**
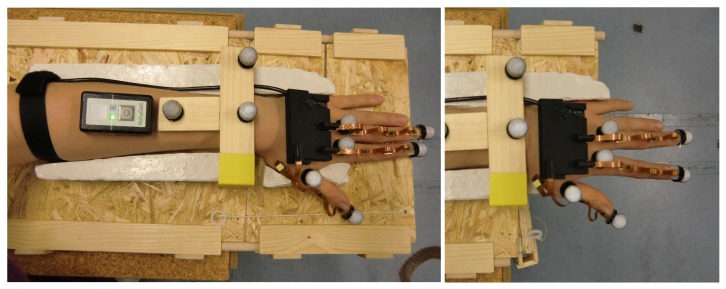
Measurement setup and positioning of the optical markers. The wooden fixture assures repeatability of the experiments and unrestricted finger motion without any translation of the forearm. The markers on top of the MCP joints are used for visualization purposes, and those on top of the fixture are used for coordinate transformation between the inertial system and the optical system.

**Figure 8:**
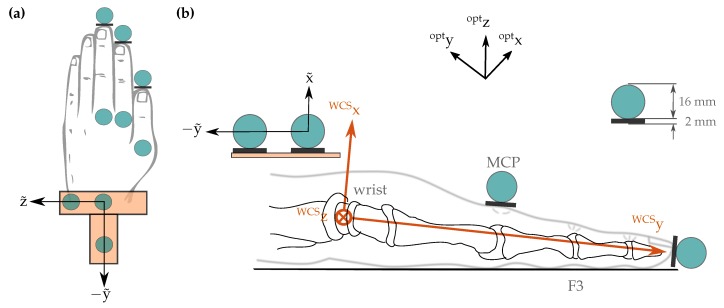
Position of the optical markers visualized as cyan circles, and definition of coordinate systems illustrated in (**a**) top view of wrist and hand, and (**b**) side view of a cut through the middle finger F3 (opt: optical system, WCS: wrist coordinate system, tilde: marker coordinate system).

The utilized optical motion capture system (Vicon Motion Systems Ltd., Oxford, UK) consists of eight infrared cameras equally distributed along the outer edge of the ceiling in a measurement chamber. We use spherical optical markers with a diameter of 16 mm, as pictured in Figure 7 and Figure 8. The motion capture system uses triangulation to measure a time series of positions in 3D space for each optical marker. The measurements were performed in the center of the measurement chamber with an average camera distance of 3 m. For this setup, the average marker position tracking error was found to be below 1 mm.

To improve the repeatability of the experiments and to minimize the effect of forearm motion, we use the setup shown in Figure 7. It consists of a plaster mold for the left arm and hand that is fixed to a wooden base. When placed in the mold, the hand is in the initial neutral pose. The abduction angle of the wrist is zero, and the thumb is abducted in a fixed angle that assures that all bones of the thumb are aligned. The L-shaped front part of the wooden base can be hinged down in a way that the palm remains supported by the base but the fingers (including the thumb) can be moved. The fixture prevents any translation of the forearm that would distort the optical measurement.

Optical markers are placed at the tips of all investigated fingers and, for visualization purposes, on top of the corresponding (T-)MCP joints, as shown in Figure 7 and Figure 8. Furthermore, three stationary markers on top of the measurement fixture facilitate coordinate transformation between the coordinate frames of the inertial sensor system and the optical motion capture system.

#### 3.1.2. Alignment of the Coordinate Frames between IMUs and Optical System

Recall that the WCS has its origin in the center of rotation of the wrist c and is aligned with the longitudinal axis of the fingers at the neutral pose. As shown in Figure 8, this directly yields the rotation ${}_{\mathrm{WCS}}^{\mathrm{opt}}q$ between the WCS and the optical coordinate system. However, we also need the translational offset between both coordinate systems, i.e., we must identify the optical system’s coordinates of the joint center. To this end, we record flexion/extension and ulnar/radial deviation movements of the wrist while all other joints are stiffened voluntary by the participant. This recording yields *n* marker position samples ${}_{\mathrm{opt}}^{F3d}p_{\mathrm{OPT}}\left(t\right)$ of the tip of F3, where the right subscript OPT denotes the measurement with the optical system. These *n* samples are used to identify the center ${}_{\mathrm{opt}}c$ of rotation of the wrist in the global coordinate frame of the optical system (opt) via the optimization problem

(28) $${}_{\mathrm{opt}}c=\underset{c\in R^{3}}{arg min}\sum_{h=1}^{n}|∥\;{}_{\mathrm{opt}}^{F3d}p_{\mathrm{OPT}}\left(t_{0}+ht_{s}\right)-c∥-\frac{1}{n}\sum_{k=1}^{n}∥\;{}_{\mathrm{opt}}^{F3d}p_{\mathrm{OPT}}\left(t_{0}+kt_{s}\right)-c∥|.$$

The function minimizes the deviations of the individual distances to c from the mean distance. A coordinate transformation consisting of the calculated translational offset ${}_{\mathrm{opt}}c$ between the measurement systems and a subsequent rotation ${}_{\mathrm{WCS}}^{\mathrm{opt}}q$ yields the marker positions in the wrist coordinate system of the inertial system:

(29) $$\begin{array}{c} {}_{\mathrm{WCS}}^{Fid}p_{\mathrm{OPT}}=L(\;{}_{\mathrm{opt}}^{Fid}p\;,\;\left(\;{}_{\mathrm{WCS}}^{\mathrm{opt}}q+\epsilon 0\right)\left(0+\epsilon (0-{}_{\mathrm{opt}}c)\right)\;) \end{array}$$

with $i\in \left\{1,2,3\right\}$. Finally, to achieve time synchronization between both measurement systems, we let each recording start with a characteristic motion of one finger, which we exploit to determine the time offset.

#### 3.1.3. Conducted Experiments

Table 4 summarizes the motions used for validation. Since any functional or complex hand motion quickly leads to occlusion or swapping of optical markers, we use pure abduction/adduction (abbreviated as A in Table 4), pure flexion/extension motions (F) and a combination of both (AF) for fingers F1, F2, and F3. The abduction of F1 stands for a planar movement of the thumb, while the combination of flexion and abduction describes the opposition of the thumb to the palm. Each experiment consisted of 10–15 repetitions of the respective movement and lasted 30–50 s. All motions were performed at a comparable speed to motions caused by neuromuscular stimulation, i.e., angular rates of the finger joints remained below 2 rad/s and contained only frequencies below 2 Hz (cf. [48]).

For the analysis, we compare the fingertip positions obtained by the inertial methods B, M1, and M2 to the reference measurements obtained from the optical motion capture system. For each measured finger $i\in \left\{1,2,3\right\}$ and each time step $t\in [t_{0}+t_{s},t_{0}+mt_{s}]$, we calculate the error

(30) $$E_{i}\left(t\right)=∥{}_{\mathrm{WCS}}^{Fid}p_{\mathrm{IMU}}\left(t\right)-{}_{\mathrm{WCS}}^{Fid}p_{\mathrm{OPT}}\left(t\right)∥,$$

and for each experiment the root-mean-square error is calculated over all *m* time steps:

(31) $${\mathrm{RMSE}}_{i}=\sqrt{\frac{1}{m}\sum_{k=1}^{m}E_{i}^{2}\left(t_{0}+kt_{s}\right)}.$$

### 3.2. Evaluation under Realistic Conditions Exploiting Characteristic Hand Poses (Setting 2)

#### 3.2.1. Setup

**Figure 9:**
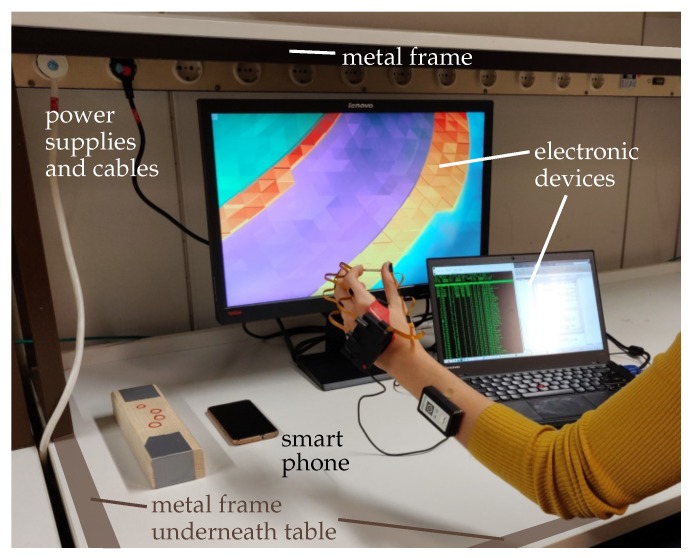
Measurement setup for Setting 2 in the direct presence of ferromagnetic materials and electronic devices.

Beyond the validation of accuracy in optical motion capture laboratories, it is likewise important to evaluate the hand motion tracking system under realistic, clinically relevant conditions. During therapy in clinics or at home, patients are indoors, and they interfere with or move in the proximity of furniture or objects containing iron or other ferromagnetic material. Hence, a homogeneous magnetic field cannot be assumed. All experiments in Setting 2 are conducted in the direct proximity of furniture containing ferromagnetic materials and electronic devices emitting electromagnetic fields, such as tables with metal legs and computers as seen in Figure 9. Moreover, complex and functional motions of the hand such as grasping or pinching are considered, since those movements are performed in motor rehabilitation training. The validation with multiple participants further allows statements on the inter-/intra-subject reliability.

#### 3.2.2. Conducted Experiments

**Figure 10:**
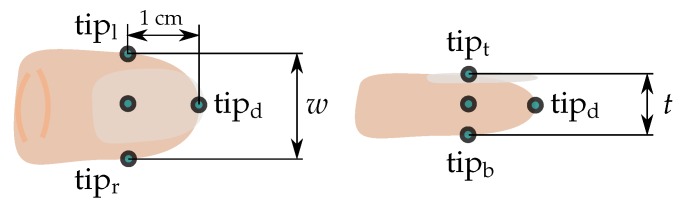
Fingertip in top view (**left**) and side view (**right**). Marked are the points of contact on the left and right (${\mathrm{tip}}_{l},{\mathrm{tip}}_{r}$), on the top and bottom (${\mathrm{tip}}_{t},{\mathrm{tip}}_{b}$) and at the distal tip (${\mathrm{tip}}_{d}$) of the finger. The parameter *w* is the width, *t* the thickness of the finger.

**Figure 11:**
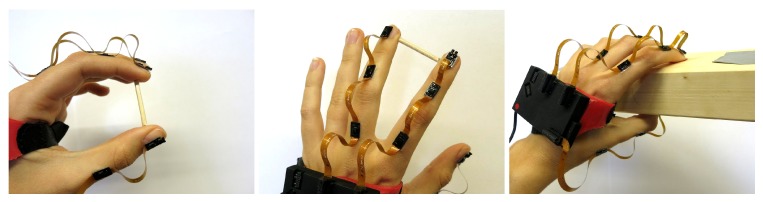
Experimental setup for the evaluation under realistic conditions. The images display the hand poses with the spacer and wooden block.

Note that such motions cannot be performed in an optical motion capture laboratory without causing repeated occlusion of the markers. Therefore, we must employ other strategies to find a ground truth for evaluation of the results: we define different points of contact around the fingertip (see Figure 10) and use characteristic hand poses and motions during which the distances between these points are constant and known. All performed experiments are summarized in Table 5. For experiments P1 and P2, we use a spacer of known length (3 cm) pinched between the bottom sides of the fingertips in the case of pair F1–F2 and between the left and right point of contact in the case of F2–F3, as illustrated in Figure 11. The fingers as well as the entire hand are moved with translation and rotation into all directions but with the spacer remaining pinched.

In the experiment P3, a pinching grip is performed with the thumb F1 and the index finger F2 such that the distal tips (tip${}_{d}$) are in contact. The pinch grip is closed and opened twice. Experiments P1 to P3 are conducted twice, and every trial lasts approximately 15–35 s. For experiment P4, we utilize a wooden block that is grasped in a way that the positions of the fingertips on the surface of the block are known. The block is moved via translation and rotation into all directions. Figure 11 illustrates the experimental setup.

For the analysis, we define the error for each time step *t* as

(32) $$E_{i}\left(t\right)=|∥{}_{\mathrm{WCS}}^{T1,\mathrm{Fi}}p_{\mathrm{IMU}}\left(t\right)-{}_{\mathrm{WCS}}^{T2,\mathrm{Fi}}p_{\mathrm{IMU}}\left(t\right)∥-d_{s}|$$

with T1, T2 $\in \{{\mathrm{tip}}_{l},{\mathrm{tip}}_{r},{\mathrm{tip}}_{b},{\mathrm{tip}}_{d}\}$, i∈{1,2,3} and s∈{P1,P2,P3,P4}. For each trial, we determine the root-mean-square error according to Equation (31).

## 4. Results

### 4.1. Results under Idealistic Conditions (Setting 1)

**Figure 12:**
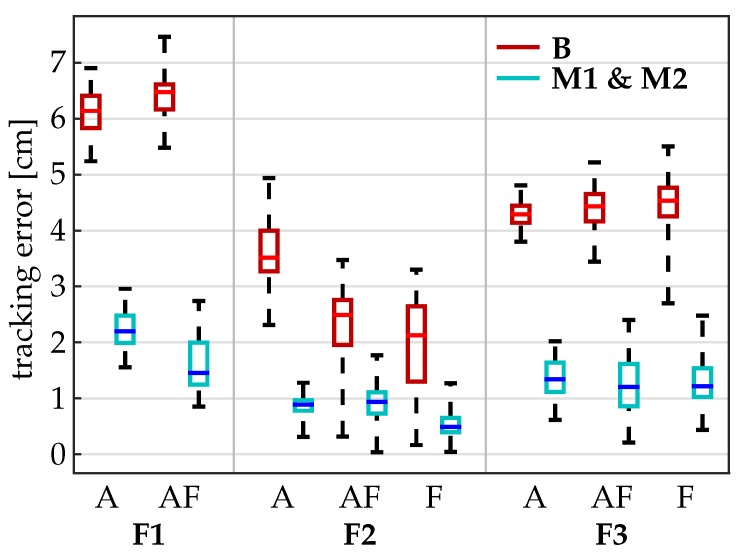
Median, 25th and 75th percentile and maximum values of the error *E* between the optical motion capture measurement and the hand sensor system with method B (red) and M1 and M2 (blue) for all conducted experiments with the volunteer in Setting 1. For a better overview, methods M1 and M2 were summarized, whereby the respective higher value was illustrated. Box-plots were calculated over time intervals of 30 seconds with at least 10 repetitions for each movement. Abbreviations: A: abduction, F: flexion, AF: abduction and flexion motion, F1: thumb, F2: index finger, F3: middle finger.

The results of Setting 1 for individual fingers and motions are presented in Table 6, and box plots for each finger and motion are displayed in Figure 12. The baseline method B yields root-mean-square tracking errors between 2 cm and 6.3 cm for the different motions and fingers. In contrast, the RMSE for the advanced methods M1 and M2 range between 0.5 cm and 1.6 cm for fingers F2 and F3 and between 1.6 cm and 2.2 cm for the anatomically more complex thumb (F1). Even in the peak values, the tracking error never exceeds 2.5 cm for F2 and F3 and 3 cm for F1. The methods M1 and M2 are found to yield very similar results, which is not surprising since no magnetic disturbances were present.

**Figure 13:**
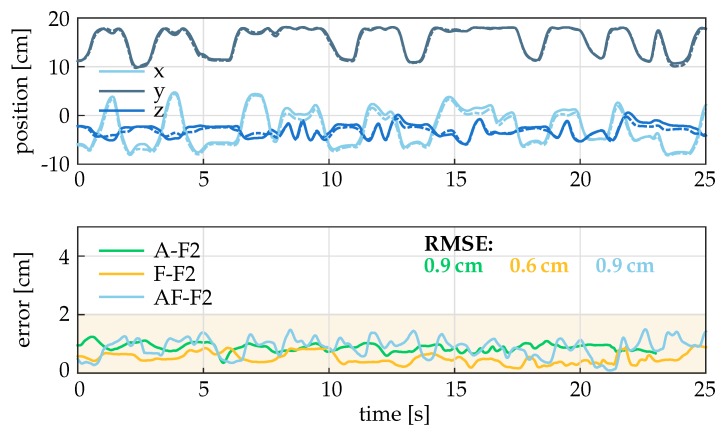
Top: Time series of x-, y-, and z-component of the fingertip position p in the WCS for the combined abduction and flexion motion of the index finger (AF-F2). The solid lines are the calculated position of the hand sensor system with method M1, the dashed lines depict the optical data. Bottom: time series of the tracking error for all three motions of the index finger F2 between the hand sensor system with method M1 and optical system. For better illustration, the signals were low-pass filtered with a cutoff frequency of 2 Hz. The error is always below the critical value of 2 cm. Abbreviations: A: abduction, F: flexion, AF: abduction and flexion motion, F2: index finger (cf. Table 4).

Exemplary time series for the estimated fingertip position (top) and the error between optical and inertial system are presented in Figure 13 for the experiments with the index finger (F2). For better differentiation between lines, the displayed data is low-pass filtered with 2 Hz, and the graphs are limited to 25 s. The upper subplot shows that the measurements with the two different systems (optical and IMU) are very close despite a broad range of motion. The lower subplot shows that the error *E* is always below 2 cm.

### 4.2. Results under Realistic Conditions (Setting 2)

**Figure 14:**
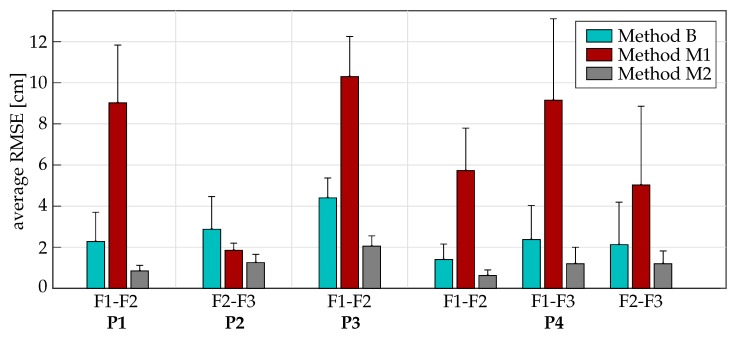
Average RMSE and standard deviations over all four participants evaluated in Setting 2 for each method. Please refer to Table 5 for a description of the experiments. Method M2 always yielded the smallest RMSE with comparatively minor differences between the participants. Abbreviations: P1–P4: experiments 1–4, F1: thumb, F2: index finger, F3: middle finger.

Average and subject-individual results for the evaluation in Setting 2 are presented in Table 7 and Figure 14 for all four experiments. All experiments are conducted in the direct proximity of furniture containing ferromagnetic materials and electronic devices emitting electromagnetic fields. It is therefore not surprising that the methods B and M1, which use magnetometer readings, are found to fail at yielding a reliable estimation of the fingertip positions. In fact, method M1 even leads to considerably larger errors in most cases than method B. Moreover, the additional errors of method B and M1 when compared to M2 vary largely from subject to subject and experiment to experiment (cf. Figure 14).

**Figure 15:**
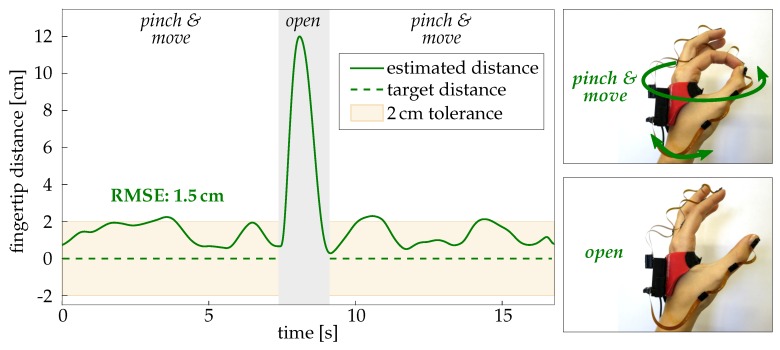
Representative time series of a pinch motion (experiment P3). The measured distance between ${\mathrm{tip}}_{d}$ of F1 and F2 (solid green line) is close to the true value (0 cm; dashed green line). Note that the pinch was released during the gray marked time period.

In contrast, the method M2, which refrains from using magnetometer readings, provided useful fingertip position estimates with RMSEs ranging between 0.5 cm and 2 cm on average. A comparison of these values with the results of Setting 1 reveals no notable differences. This means that the proposed method M2 provides accurate measurements not only under idealistic conditions but also in a far more realistic setting and for more complex functional motions. Figure 15 shows a representative time series with method M2 of one trial of experiment P3 (pinch of thumb and index finger while moving the hand arbitrarily in space). In this case, the distance between the fingertip position ${\mathrm{tip}}_{d}$ is slightly overestimated, and the positive offset contributes most to the RMSE value.

## 5. Discussion

We proposed and evaluated an IMU-based hand sensor system and a set of novel quaternion-based methods for the estimation of the fingertip positions. Unlike gloves, the proposed system preserves the sense of touch. The presented methods do not rely on complex calibration procedures or motions that must be performed precisely, both of which are challenging for patients with motor-impaired hands. Instead, our methods use only a single initial pose. The sensor system was evaluated with able-bodied subjects in two different validation settings: (1) in an optical motion capture laboratory as well as (2) in a realistic setting. In the first setting, motions were performed in an ideal magnetic environment, and the positions measured with the proposed system were compared with positions recorded by a marker-based optical reference system. The accuracy of the reference system itself is limited by skin (and thus markers) moving relative to the bone and by inaccuracies of marker placement. In the second setting, motions were performed in the presence of severe magnetic disturbances, and the aforementioned limitations are overcome by using functional movements with known fingertip distances.

The precision of the proposed methods under laboratory conditions is comparable to the precision reported in the literature. There are several previous contributions for IMU-based hand motion tracking that use customized rigid measurement platforms [24,25] or blocks of wood cut to specific angles [23] instead of considering measurements in human hands. Connolly et al. perform experiments in patients but present only coefficients of variation based on video recordings [23]. To the best of our knowledge, only two previous research studies evaluated kinematic measurements (angles or positions) in human subjects. These are Kortier et al. [19] and van den Noort et al. [20]. In both studies, the proposed system is compared to optical measurements recorded in laboratory environments. While Kortier et al. observe mean errors of 0.5 cm for a pure flexion/extension motion of F2 and 1.2 cm for a circular motion of F2, van den Noort et al. found deviations of 1–2 cm for the latter. Our experiments show similar results with 0.5 cm for the pure flexion/extension motion and 0.9 cm for the combined abduction and flexion motion of F2 under idealistic laboratory conditions.

In contrast to previous contributions, we additionally evaluated our methods in a second setup containing magnetic disturbances as they would occur in many application scenarios. We found that the two methods relying on magnetometer measurements (B and M1) are not reliable under these conditions, with RMSE of up to 15 cm (cf. Table 7, subject #1). Thus, for practical applications in realistic settings, we prefer method M2, which achieves average RMSEs below 1.4 cm for the index and middle fingertip and below 2.1 cm for the thumb tip in all trials of all four evaluated subjects.

While the observed deviations of approximately 1–2 cm for the best method M2 are small, it is important to discuss potential sources of these remaining deviations. We investigate error propagation using a Simscape Multibody implementation (Matlab/Simulink) of the biomechanical hand model and consider the following error sources: (1) Functional segment length ratios vary by a few percents from subject to subject, as suggested by literature on hand anthropometry (cf. Section 2.2). We find that using fixed ratios can lead to fingertip position errors of a few millimeters. (2) Similarly small errors may occur if sensor orientation estimates exhibit errors of a few degrees. (3) An analysis of the angles that are neglected by the application of joint constraints (methods M1 and M2, cf. Figure 6) yields average absolute values between 5 and 10 degrees. For segment lengths in the range of several centimeters, such angles are found to affect the measurement results by approximately half a centimeter. (4) Likewise, we expect inaccuracies in the sensor-to-segment alignment to be in the order of 5 to 10 degrees, which entails the same order of error magnitude. However, alignment of the thumb (F1) axes is slightly more challenging, which might explain the slightly larger deviations found for F1. We conclude that the aforementioned remaining deviations of approximately 1–2 cm are most likely due to a combination of the two latter aspects. Further investigations might yield deeper insights.

Method M2 completely avoids the use of magnetometer readings. Absolute heading information and a gyroscope bias estimation are obtained from the initial neutral pose. However, even the best IMUs exhibit some random bias instability, which suggests that the accuracy of Method M2 will deteriorate with time and the fingertip positions will eventually become inaccurate. Hence, future studies should include a larger number of subjects and trials and evaluate the long-time stability of Method M2. A promising direction of future research is to combine the method with recently proposed approaches that exploit kinematic constraints to compensate heading drift in magnetometer-free sensor fusion completely [49,50,51,52].

## 6. Conclusions

The hand sensor system is designed to be part of a feedback-controlled hand neuroprosthesis for the rehabilitation of patients who suffer from a motor impairment of the hand (e.g., after stroke). Therefore, the estimation accuracy has to match the sensitivity of the neuromuscular stimulation. The most advanced current systems are capable of generating coarse motions such as opening and closing the hand to grasp large objects. The low sensitivity and selectivity of stimulation through surface electrodes do not allow control of individual fingers to the exact centimeter. We conclude that the achieved accuracy (RMSE approx. 1 cm) is sufficient for feedback control of the neuroprosthesis. We have recently successfully applied the proposed hand sensor system for the specific purpose of finding optimal stimulation points in electrode arrays [11].

In the future, the hand sensor system will be combined with fingertip force sensors that detect contact with an object and thus yield additional information when the patient grasps and holds an object [53]. Future work should also aim at a reduction of the sensor system’s weight and dimensions, to further reduce the impact of the system on movements with the motor-impaired hand. In addition, if sufficient miniaturization of wireless IMUs is achieved in the future, the hand sensor system will no longer require flexible PCBs between the sensor units. Finally, the use of higher sampling rates to facilitate highly accurate motion tracking of faster and less smooth motions might be advisable, especially in non-clinical application domains. The resulting challenges of large wireless communication load and accidental swapping of sensor units might be addressed by recently developed methods for event-based communication [54].

## Figures and Tables

**Table 1:** Overview of suggested hand motion tracking systems for clinical applications. This list does not claim to be complete and rather shows examples. The advantages and disadvantages refer to the use in closed-loop functional electrical stimulation for grasping.

System Type	Examples	Advantages and Disadvantages
Optical systems with markers	Vicon (*Vicon Motion Systems Ltd.,* *Oxford, UK*)	(+) accurate (–) extensive setup by expert, expensive, line-of-sight restriction, stationary
Optical systems without markers	Kinect V2 (*Microsoft, Redmond, WA,* *USA*), Leap Motion (*Leap Motion, San Francisco, CA, USA*)	(+) contactless, affordable (–) limited accuracy, line-of-sight restriction
Sensor gloves with bend sensors	5DT Data Glove Ultra (*5DT Inc.,* *Orlando, FL, USA*), Cyperglove III (*CyberGlove Systems Inc. LLC, San* *Jose, CA, USA*)	(+) quick setup (–) less sense of touch, glove not suitable for spastic hand, hygienically problematic, measures only angles (no accelerations/ velocities/positions)
Sensor gloves with IMUs	IGS Cobra Glove (*Synertial, Lewes,* *UK*), PowerGlove [19,20]	(+) quick setup, detailed measurements (–) less sense of touch, glove not suitable for spastic hand, hygienically problematic, uses magnetometers and calibration motions

**Table 2:** Ratios of the functional lengths of the proximal ($l_{p}$), middle ($l_{m}$) and distal ($l_{d}$) phalanges according to Hamilton et al. [40] (F2–F5), Buchholz et al. and Buryanov et al. [39,42] (F1). The 95% confidence intervals (CI) observed by [40] are also included.

	F1	F2		F3		F4		F5	
	$l_{p}/l_{d}$	$l_{p}/l_{m}$	$l_{m}/l_{d}$	$l_{p}/l_{m}$	$l_{m}/l_{d}$	$l_{p}/l_{m}$	$l_{m}/l_{d}$	$l_{p}/l_{m}$	$l_{m}/l_{d}$
Ratios	0.98	1.86	1.24	1.72	1.36	1.70	1.29	1.91	1.06
95% CI		0.018	0.018	0.013	0.016	0.016	0.016	0.022	0.022

**Table 3:** Thickness of soft tissue at the fingertip in mm (mean ± standard deviation) according to Buryanov et al. [42].

F1	F2	F3	F4	F5
5.67±0.61	3.84±0.59	3.95±0.61	3.95±0.60	3.73±0.62

**Table 4:** Overview of experiments in Setting 1 with optical reference (A: abduction, F: flexion, AF: abduction and flexion).

Experiment ID	Description
A-F1, A-F2, A-F3	Pure abduction motion of F1, F2, F3
F-F2, F-F3	Pure flexion motion of F2 and F3
AF-F1, AF-F2, AF-F3	Combined abduction and flexion motion of F1, F2, F3

**Table 5:** Overview of experiments in Setting 2 with predefined postures in a magnetically disturbed environment.

Experiment ID	Description
P1	Spacer with length $d_{P1}=3\mathrm{cm}$ between F1 ${\mathrm{tip}}_{b}$ and F2 ${\mathrm{tip}}_{b}$
P2	Spacer with length $d_{P2}=3\mathrm{cm}$ between F2 ${\mathrm{tip}}_{l}$ and F3 ${\mathrm{tip}}_{r}$
P3	F1 tip${}_{d}$ and F2 tip${}_{d}$ in contact, distance $d_{P3}=0\mathrm{cm}$
P4	All fingers fixed on a wooden block that is moved in space, distance $d_{P4}$ predefined for each pair of fingers ($d_{P4,F1F2}=5.3\mathrm{cm}$, $d_{P4,F1F3}=4.6\mathrm{cm}$, $d_{P4,F2F3}=2.5\mathrm{cm}$)

**Table 6:** Mean and standard deviation (std) of error *E* [cm] and RMSE [cm] for the evaluation of methods B, M1, and M2 when compared against an optical motion capture system in Setting 1 for Subject #1. Abbreviations: A: abduction, F: flexion, AF: abduction and flexion motion (cf. Table 4).

Experiment ID	Method B		Method M1		Method M2	
	Mean±Std(*E*)	RMSE	Mean±Std(*E*)	RMSE	Mean±Std(*E*)	RMSE
A-F1	6.0±0.60	6.1	2.2±0.36	2.2	1.8±0.18	1.8
A-F2	3.6±0.47	3.6	0.9±0.14	0.9	0.9±0.19	1.0
A-F3	4.2±0.44	4.3	1.3±0.26	1.4	0.6±0.17	0.7
AF-F1	6.3±0.52	6.3	1.6±0.45	1.6	2.0±0.52	2.1
AF-F2	2.2±0.63	2.3	0.9±0.30	0.9	0.9±0.49	1.0
AF-F3	4.3±0.48	4.4	1.2±0.45	1.3	0.9±0.45	1.0
F-F2	2.0±0.75	2.1	0.5±0.20	0.6	0.6±0.19	0.6
F-F3	4.3±0.76	4.4	1.2±0.34	1.3	0.8±0.34	0.8

**Table 7:** Mean and standard deviation (std) of error *E* [cm] and RMSE [cm] for experiments P1–P4 in the realistic environment of Setting 2. Average and individual results of each participant are included.

Experiment	Fingers	Subject	Method B		Method M1		Method M2	
ID			Mean±Std(*E*)	RMSE	Mean±Std(*E*)	RMSE	Mean±Std(*E*)	RMSE
P1	F1–F2	#1	3.3±1.51	3.7	6.7±1.85	7.0	0.8±0.57	1.0
		#2	3.5±1.14	3.7	12.6±1.24	12.7	0.4±0.25	0.5
		#3	0.8±0.47	0.9	10.6±1.74	10.7	1.2±0.24	1.2
		#4	0.7±0.45	0.8	5.1±2.67	5.7	0.6±0.35	0.7
		**Average**	**2.1±0.89**	**2.3**	**8.8±1.88**	**9.0**	**0.8±0.35**	**0.9**
P2	F2–F3	#1	4.9±1.97	5.3	1.8±1.27	2.2	1.7±0.65	1.8
		#2	0.9±0.68	1.1	1.1±1.36	1.8	0.6±0.27	0.7
		#3	3.0±1.14	3.2	1.8±1.22	2.1	1.0±0.39	1.1
		#4	1.8±0.45	1.9	1.1±0.59	1.3	1.4±0.44	1.4
		**Average**	**2.7±1.06**	**2.9**	**1.5±1.11**	**1.9**	**1.2±0.44**	**1.3**
P3	F1–F2	#1	4.5±3.17	5.5	11.0±2.61	11.3	1.4±0.55	1.5
		#2	4.1±1.19	4.3	12.5±2.83	12.8	1.6±0.42	1.6
		#3	4.6±1.47	4.9	9.2±2.34	9.5	2.6±0.41	2.6
		#4	2.7±1.05	2.9	7.1±2.69	7.6	2.5±0.44	2.5
		**Average**	**4.0±1.72**	**4.4**	**10.0±2.62**	**10.3**	**2.0±0.46**	**2.0**
P4	F1–F2	#1	1.9±1.59	2.5	4.0±2.51	4.7	0.7±0.39	0.8
		#2	0.5±0.40	0.7	8.0±3.48	8.8	0.2±0.14	0.2
		#3	0.5±0.39	0.7	5.5±2.73	6.2	0.5±0.32	0.6
		#4	1.6±0.56	1.7	2.9±1.28	3.2	0.9±0.24	0.9
		**Average**	**1.1±0.74**	**1.4**	**5.1±2.5**	**5.7**	**0.6±0.27**	**0.6**
P4	F1–F3	#1	4.8±1.97	5.2	15.0±2.52	15.2	0.4±0.23	0.4
		#2	0.9±0.51	1.0	9.3±3.28	9.9	1.9±0.26	2.0
		#3	1.2±0.85	1.5	8.6±3.59	9.4	2.0±0.32	2.0
		#4	1.5±0.89	1.8	3.8±1.66	4.1	0.4±0.19	0.4
		**Average**	**2.1±1.06**	**2.4**	**9.2±2.76**	**9.7**	**1.2±0.25**	**1.2**
P4	F2–F3	#1	5.5±1.55	5.7	11.0±2.47	11.3	0.4±0.40	0.6
		#2	0.5±0.48	0.7	3.9±1.54	4.2	1.9±0.47	2.0
		#3	0.9±0.54	1.0	2.8±2.48	3.7	1.6±0.48	1.6
		#4	0.9±0.60	1.1	0.8±0.43	0.9	0.5±0.32	0.6
		**Average**	**2.0±0.79**	**2.1**	**4.6±1.73**	**5.0**	**1.1±0.42**	**1.2**

## Abbreviations

The following abbreviations are used in this manuscript:

A	Abduction
CI	Confidence interval
DIP	Distal interphalangeal joint
DoF	Degrees of freedom
F	Flexion
F1	Finger no. 1 (thumb)
F2	Finger no. 2 (index)
F3	Finger no. 3 (middle)
F4	Finger no. 4 (ring)
F5	Finger no. 5 (little)
FES	Functional electrical stimulation
IMU	Inertial measurement unit
ipa	Initial pose aligned
ISB	International Society of Biomechanics
MCP	Metacarpal-phalangeal joints
P1…4	Experiments P1 to P4 in setting 2
PIP	Proximal interphalangeal joint
RMSE	Root mean square error
SCI	Spinal cord injury
std	Standard deviation
T-CMC	Thumb-carpo-metacarpal joint
T-IP	Thumb-interphalangeal joint
T-MCP	Thumb-metacarpal-phalangeal joint
WCS	Wrist coordinate system

## References

[1] Peckham P.H., Knutson J.S. (2005). Functional Electrical Stimulation for Neuromuscular Applications. Annu. Rev. Biomed. Eng..

[2] Soska A., Freeman C., Rogers E. ILC for FES-based Stroke Rehabilitation of Hand and Wrist. Proceedings of the 2012 IEEE International Symposium on Intelligent Control.

[3] Schauer T. (2017). Sensing motion and muscle activity for feedback control of functional electrical stimulation: Ten years of experience in Berlin. Annu. Rev. Control.

[4] Valtin M., Seel T., Raisch J., Schauer T. Iterative learning control of drop foot stimulation with array electrodes for selective muscle activation. Proceedings of the Preprints 19th WC IFAC.

[5] Müller P., Balligand C., Seel T., Schauer T. (2017). Iterative Learning Control and System Identification of the Antagonistic Knee Muscle Complex During Gait Using Functional Electrical Stimulation. IFAC-PapersOnLine.

[6] Freeman C., Hughes A.M., Burridge J., Chappell P., Lewin P., Rogers E. (2009). Iterative learning control of FES applied to the upper extremity for rehabilitation. Control Eng. Pract..

[7] Freeman C. (2015). Control System Design for Electrical Stimulation in Upper Limb Rehabilitation: Modelling, Identification and Robust Performance.

[8] Passon A., Seel T., Massmann J., Freeman C., Schauer T. Iterative learning vector field for FES-supported cyclic upper limb movements in combination with robotic weight compensation. Proceedings of the 2018 IEEE/RSJ International Conference on Intelligent Robots and Systems.

[9] Popovic M.R., Popovic D.B., Keller T. (2002). Neuroprostheses for grasping. Neurol. Res..

[10] Koutsou A.D., Moreno J.C., del Ama A.J., Rocon E., Pons J.L. (2016). Advances in selective activation of muscles for non-invasive motor neuroprostheses. J. Neuroeng. Rehabil..

[11] Salchow-Hömmen C., Jankowski N., Valtin M., Schönijahn L., Böttcher S., Dähne F., Schauer T. (2018). User-centered practicability analysis of two identification strategies in electrode arrays for FES induced hand motion in early stroke rehabilitation. J. Neuroeng. Rehabil..

[12] Colombo R., Sanguineti V. (2018). Rehabilitation Robotics: Technology and Application.

[13] De Vries W.H., Veeger H.E., Baten C.T., van der Helm F.C. (2009). Magnetic distortion in motion labs, implications for validating inertial magnetic sensors. Gait Posture.

[14] Seel T., Ruppin S. (2017). Eliminating the Effect of Magnetic Disturbances on the Inclination Estimates of Inertial Sensors. IFAC-PapersOnLine.

[15] Erol A., Bebis G., Nicolescu M., Boyle R.D., Twombly X. (2007). Vision-based hand pose estimation: A review. Comput. Vis. Image Underst..

[16] Dipietro L., Sabatini A.M., Dario P. (2008). A survey of glove-based systems and their applications. IEEE Trans. Syst. Man Cybern. C.

[17] Fahn C.S., Sun H. (2010). Development of a Fingertip Glove Equipped with Magnetic Tracking Sensors. Sensors.

[18] Saggio G., Bocchetti S., Pinto C.A., Orengo G., Giannini F. A novel application method for wearable bend sensors. Proceedings of the 2nd International Symposium on Applied Sciences in Biomedical and Communication Technologies.

[19] Kortier H.G., Sluiter V.I., Roetenberg D., Veltink P.H. (2014). Assessment of hand kinematics using inertial and magnetic sensors. J. Neuroeng. Rehabil..

[20] Van den Noort J.C., Kortier H.G., van Beek N., Veeger D.H.E.J., Veltink P.H., Bensmaia S.J. (2016). Measuring 3D Hand and Finger Kinematics—A Comparison between Inertial Sensing and an Opto-Electronic Marker System. PLoS ONE.

[21] Westerveld A.J., Kuck A., Schouten A.C., Veltink P.H., van der Kooij H. Grasp and release with surface functional electrical stimulation using a Model Predictive Control approach. Proceedings of the 2012 Annual International Conference of the IEEE Engineering in Medicine and Biology Society.

[22] Kim Y.S., Soh B.S., Lee S.G. (2005). A new wearable input device: SCURRY. IEEE Trans. Ind. Electron..

[23] Connolly J., Condell J., O’Flynn B., Sanchez J.T., Gardiner P. (2018). IMU Sensor-Based Electronic Goniometric Glove for Clinical Finger Movement Analysis. IEEE Sens. J..

[24] Choi Y., Yoo K., Kang S.J., Seo B., Kim S.K. (2018). Development of a low-cost wearable sensing glove with multiple inertial sensors and a light and fast orientation estimation algorithm. J. Supercomput..

[25] Lin B.S., Lee I., Yang S.Y., Lo Y.C., Lee J., Chen J.L. (2018). Design of an Inertial-Sensor-Based Data Glove for Hand Function Evaluation. Sensors.

[26] Salchow C., Valtin M., Seel T., Schauer T. Development of a Feedback-Controlled Hand Neuroprosthesis: FES-Supported Mirror Training. Proceedings of the AUTOMED Workshop.

[27] Valtin M., Salchow C., Seel T., Laidig D., Schauer T. (2017). Modular finger and hand motion capturing system based on inertial and magnetic sensors. Curr. Dir. Biomed. Eng..

[28] Salchow-Hömmen C., Thomas T., Valtin M., Schauer T. (2018). Automatic control of grasping strength for functional electrical stimulation in forearm movements via electrode arrays. at-Autom.

[29] Clauser C.E., McConville J.T., Young J.W. (1969). Weight, Volume, and Center of Mass of Segments of the Human Body (AMRL TR 69-70).

[30] Zhang R., Hoflinger F., Reind L.M. (2014). Calibration of an IMU using 3-D rotation platform. IEEE Sens. J..

[31] Winter D.A. (2009). Biomechanics and Motor Control of Human Movement.

[32] Cobos S., Ferre M., Uran M.S., Ortego J., Pena C. Efficient human hand kinematics for manipulation tasks. Proceedings of the 2008 IEEE/RSJ International Conference on Intelligent Robots and Systems.

[33] Cobos S., Ferre M., Aracil R., Ortego J., Angel M. (2010). Simplified Human Hand Models for Manipulation Tasks. Cutting Edge Robotics 2010.

[34] Cocchiarella D.M., Kociolek A.M., Tse C.T.F., Keir P.J. (2016). Toward a realistic optoelectronic-based kinematic model of the hand: Representing the transverse metacarpal arch reduces accessory rotations of the metacarpophalangeal joints. Comput. Methods Biomech. Biomed. Eng..

[35] Keir P., Cocciarella D., Kociolek A. Development of a kinematic hand model with a realistic representation of the metacarpal arch. Proceedings of the 24th ISB Congress.

[36] Wu G., van der Helm F.C., Veeger H., Makhsous M., van Roy P., Anglin C., Nagels J., Karduna A.R., McQuade K., Wang X. (2005). ISB recommendation on definitions of joint coordinate systems of various joints for the reporting of human joint motion–Part II: Shoulder, elbow, wrist and hand. J. Biomech..

[37] Goislard de Monsabert B., Visser J., Vigouroux L., van der Helm F., Veeger H. (2014). Comparison of three local frame definitions for the kinematic analysis of the fingers and the wrist. J. Biomech..

[38] Gustus A., Stillfried G., Visser J., Jörntell H., van der Smagt P. (2012). Human hand modelling: Kinematics, dynamics, applications. Biol. Cybernet..

[39] Buchholz B., Armstrong T.J., Goldstein S.A. (1992). Anthropometric data for describing the kinematics of the human hand. Ergonomics.

[40] Hamilton R., Dunsmuir R.A. (2002). Radiographic Assessment of the Relative Lengths of the Bones of the Fingers of the Human Hand. J. Hand. Surg. Eur..

[41] Park A.E., Fernandez J.J., Schmedders K., Cohen M.S. (2003). The fibonacci sequence: Relationship to the human hand. J. Hand. Surg. Am..

[42] Buryanov A., Kotiuk V. (2010). Proportions of Hand Segments. Int. J. Morphol..

[43] Radavelli L., Simoni R., De Pieri E.R., Martins D. (2012). A Comparative Study of the Kinematics of Robots Manipulators by Denavit-Hartenberg and Dual Quaternion. Mecánica Comput. Multi-Body Syst..

[44] Leclercq G., Lefèvre P., Blohm G. (2013). 3D kinematics using dual quaternions: Theory and applications in neuroscience. Front. Behav. Neurosci..

[45] Hamilton W.R. (1844). II. On quaternions; Or on a new system of imaginaries in algebra. Philos. Mag. Ser. 3.

[46] Kenwright B. A beginners guide to dual-quaternions: What they are, how they work, and how to use them for 3D character hierarchies. Proceedings of the 20th WSCG International Conference on Computer Graphics, Visualization and Computer Vision 2012.

[47] Aumüller G., Aust G., Doll A., Engele J., Kirsch J., Mense S., Reißig D., Salvetter J., Schmidt W., Schmitz F. (2007). Anatomie.

[48] Kutlu M., Freeman C., Hughes A.M., Spraggs M. (2017). A Home-based FES System for Upper-limb Stroke Rehabilitation with Iterative Learning Control. IFAC-PapersOnLine.

[49] Kok M., Hol J.D., Schön T.B. An optimization-based approach to human body motion capture using inertial sensors. Proceedings of the 19th IFAC World Congress.

[50] Müller P., Bégin M.A., Schauer T., Seel T. (2017). Alignment-Free, Self-Calibrating Elbow Angles Measurement using Inertial Sensors. IEEE J. Biomed. Health Inform..

[51] Laidig D., Schauer T., Seel T. Exploiting Kinematic Constraints to Compensate Magnetic Disturbances when Calculating Joint Angles of Approximate Hinge Joints from Orientation Estimates of Inertial Sensors. Proceedings of the 2017 International Conference on Rehabilitation Robotics (ICORR).

[52] Teufl W., Miezal M., Taetz B., Fröhlich M., Bleser G. (2018). Validity, Test-Retest Reliability and Long-Term Stability of Magnetometer Free Inertial Sensor Based 3D Joint Kinematics. Sensors.

[53] Kortier H.G., Schepers H.M., Veltink P.H. (2016). Identification of Object Dynamics Using Hand Worn Motion and Force Sensors. Sensors.

[54] Laidig D., Trimpe S., Seel T. (2016). Event-Based Sampling for Reducing Communication Load in Realtime Human Motion Analysis by Wireless Inertial Sensor Networks. Curr. Dir. Biomed. Eng..

